# A phase II, open-label, multicentre study to evaluate the immunogenicity and safety of an adjuvanted prepandemic (H5N1) influenza vaccine in healthy Japanese adults

**DOI:** 10.1186/1471-2334-10-338

**Published:** 2010-11-25

**Authors:** Hideaki Nagai, Hideyuki Ikematsu, Kazuyoshi Tenjinbaru, Atsushi Maeda, Mamadou Dramé, François P Roman

**Affiliations:** 1 National Hospital Organization Tokyo National Hospital, 3-1-1, Takeoka, Kiyose-city, Tokyo 204-8585, Japan; 2 Hara-doi Hospital, 6-40-8, Aoba, Higashi-ku, Fukuoka 813-8588, Japan; 3 Clinical Development Vaccines, GlaxoSmithKline Japan; 4 Global Clinical Research & Development, GlaxoSmithKline Biologicals, Wavre, Belgium

## Abstract

**Background:**

Promising clinical data and significant antigen-sparing have been demonstrated for a pandemic H5N1 influenza split-virion vaccine adjuvanted with AS03_A_, an α-tocopherol-containing oil-in-water emulsion-based Adjuvant System. Although studies using this formulation have been reported, there have been no data for Japanese populations. This study therefore aimed to assess the immunogenicity and tolerability of a prepandemic (H5N1) influenza vaccine adjuvanted with AS03_A _in Japanese adults.

**Methods:**

This open-label, single-group study was conducted at two centres in Japan in healthy Japanese males and females aged 20-64 years (n = 100). Subjects received two doses of vaccine, containing 3.75 μg haemagglutinin of the A/Indonesia/5/2005-like IBCDC-RG2 Clade 2.1 (H5N1) strain adjuvanted with AS03_A_, 21 days apart. The primary endpoint evaluated the humoral immune response in terms of H5N1 haemagglutination inhibition (HI) antibody titres against the vaccine strain (Clade 2.1) 21 days after the second dose. Ninety five percent confidence intervals for geometric mean titres, seroprotection, seroconversion and seropositivity rates were calculated. Secondary and exploratory endpoints included the assessment of the humoral response in terms of neutralising antibody titres, the response against additional H5N1 strains (Clade 1 and Clade 2.2), as well as the evaluation of safety and reactogenicity.

**Results:**

Robust immune responses were elicited after two doses of the prepandemic influenza vaccine adjuvanted with AS03_A_. Overall, vaccine HI seroconversion rates and seroprotection rates were 91% 21 days after the second vaccination. This fulfilled all regulatory acceptance criteria for the vaccine-homologous HI antibody level. A substantial cross-reactive humoral immune response was also observed against the virus strains A/turkey/Turkey/1/2005 (Clade 2.2) and A/Vietnam/1194/2004 (Clade 1) after the second vaccine administration. A marked post-vaccination response in terms of neutralising antibody titres was demonstrated and persistence of the immune response was observed 6 months after the first dose. The vaccine was generally well tolerated and there were no serious adverse events reported.

**Conclusions:**

The H5N1 candidate vaccine adjuvanted with AS03_A _elicited a strong and persistent immune response against the vaccine strain A/Indonesia/5/2005 in Japanese adults. Vaccination with this formulation demonstrated a clinically acceptable reactogenicity profile and did not raise any safety concerns in this population.

**Trial registration:**

Clinicaltrials.gov NCT00742885

## Background

The highly pathogenic influenza A H5N1 virus first emerged as a cause of death in poultry in 1996 and was identified in humans in 1997; 18 individuals in Hong Kong became severely ill, with six deaths reported, following contact with infected birds [1]. The H5N1 virus reappeared in 2003 and has since caused 295 deaths from 499 confirmed cases worldwide (World Health Organization [WHO] as of 08 June 2010) [2].

The WHO declared a pandemic alert stage 6 due to an outbreak of an influenza A virus (A/H1N1) on 11 June 2009. As of 13 June 2010, more than 214 countries have reported a total of at least 18,172 deaths [3]. However, the highly pathogenic H5N1 strain is also a potential pandemic virus and, therefore, it remains of great concern. The H5N1 virus currently meets two of the three criteria for a global pandemic strain: H5 is a haemagglutinin (HA) subtype against which most of the human population is virtually naïve, and the virus is able to replicate in humans causing severe disease and death [4]. To date, the virus has not acquired the ability for large-scale human-to-human transmission - although isolated cases have occurred [5,6].

Vaccination is a vital part of the strategy to mitigate morbidity and mortality caused by influenza pandemics [7] and is integral to the WHO global influenza preparedness plan [8]. Pandemic vaccines are produced as soon as a pandemic is declared using the specific pandemic viral strain. However, these vaccines will only be available several months after the onset of the pandemic due to the length of time required for their manufacture [8].

The efficacy of prepandemic vaccines, which are produced in advance of a pandemic, relies on the vaccine's ability to provide a breadth of protection against different, related strains, as it is not possible to predict exactly the strain that will cause such an outbreak in advance due to the progressive accumulation of antigenic changes.

Promising clinical data have been generated for a prepandemic split-virion influenza vaccine formulated with an α-tocopherol containing, oil-in-water (O/W) emulsion-based Adjuvant System, AS03. This vaccine has demonstrated a good safety profile in randomised clinical trials in a range of human populations [9-11]. AS03 adjuvantation of the H5N1 vaccine allows for a reduction in the amount of antigen required per dose in order to induce potentially protective immune responses in humans, and it can also induce strong cross-strain and cross-clade immunity as is required for an effective prepandemic vaccine [9,10,12,13]. The A/Vietnam/1194/2004 H5N1 strain was identified as having the potential to cause a human pandemic and was thus used in several AS03 candidate vaccine studies, leading to the initial approval of a prepandemic H5N1 vaccine (*Prepandrix*™ GSK Biologicals, Rixensart, Belgium) [9,10,12-14]. This vaccine has also been shown to protect against lethal heterologous challenge in an animal model [15]. A new emerging H5N1 strain was identified by the WHO sentinel laboratory (A/Indonesia/5/2005) in 2005 [16], which was subsequently recommended by the WHO for use in vaccines, and has also been employed as part of a potential prepandemic vaccine.

In January 2004, there were confirmed outbreaks of H5N1 infection in Japanese poultry, which led to increasing concern regarding the ability of the virus to infect humans. Following the development of a whole-virus, aluminium-adjuvanted H5N1 vaccine in 2007, the Japanese Ministry of Health, Labour and Welfare began domestic manufacture of this vaccine for stockpiling [17]. However, the immune response elicited by an aluminium-adjuvanted H5N1 vaccine indicated insufficient immunogenicity [18]. Clinical data on the use of alternative vaccines, such as the AS03-adjuvanted prepandemic H5N1 vaccine, in the Japanese population would therefore be of interest.

This open-label, single-arm study set out to evaluate the humoral immune response and safety of two doses of AS03_A_-adjuvanted A/Indonesia/5/2005 H5N1 vaccine and determine its putative clinical value as a prepandemic vaccine in healthy Japanese adults.

## Methods

### Vaccine

The A/H5N1 monovalent split-virion recombinant influenza prepandemic candidate vaccine was manufactured by GlaxoSmithKline (GSK) Biologicals in Quebec, Canada. The vaccine contained 3.75 μg HA of the A/Indonesia/5/2005-like IBCDC-RG2; Clade 2.1 (H5N1) strain (Centers for Disease Control and Prevention [CDC], Atlanta, USA) adjuvanted with AS03_A _(an O/W emulsion-based Adjuvant System containing 11.86 mg of α-tocopherol).

### Study design

This open-label, single-group study (NCT00742885) was conducted at two centres in Japan. The study set out to evaluate the humoral immune response generated by two doses of the adjuvanted prepandemic A/Indonesia/5/2005 H5N1 vaccine in terms of H5N1 haemagglutination inhibition (HI) antibody titres against the vaccine strain (Clade 2.1) 21 days after the second dose. Secondary and exploratory endpoints included the assessment of the humoral response in terms of neutralising antibody titres, the response against additional H5N1 strains (Clade 1 and Clade 2.2), as well as the evaluation of safety and reactogenicity. The latter were assessed in terms of the occurrence of solicited local and general adverse events (AEs), unsolicited AEs, serious AEs (SAEs) and by the evaluation of medically attended visits and selected laboratory parameters.

Healthy Japanese men and women aged between 20 and 64 years at the time of first vaccination were eligible for inclusion if they were in good general health and provided written informed consent before enrolment. Subjects were stratified by age (20-40 years and 41-64 years) in a 1:1 ratio. Subjects were excluded from the study if they had an axillary temperature ≥37.5°C, or acute symptoms of more than mild severity on the scheduled date of first vaccination; any confirmed or suspected immunosuppressive or immunodeficient condition including history of human immunodeficiency virus (HIV) infection; administration of any registered vaccine within 30 days before study vaccination or planned administration within the first vaccination period up to blood sampling at Day 42; use of any investigational or non-registered product (drug or vaccine) within 30 days prior to study enrolment or planned use during the study period, and history of previous H5N1 vaccination; or history of H5N1 influenza infection.

**Figure 1:**
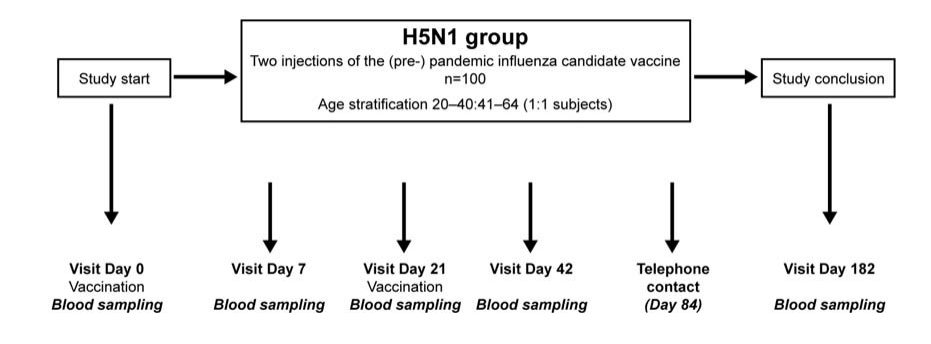
**Trial profile**.

Subjects received two doses of the prepandemic (H5N1) influenza candidate vaccine. The vaccine was administered intramuscularly in the deltoid region of the non-dominant arm on Day 0 and the second dose was given 21 days later in the non-dominant arm. Blood samples were collected for serological testing on Day 0, 7, 21, 42 and 182, and telephone contact was made on Day 84 to record any unsolicited AEs (Figure 1). Solicited AEs were assessed up to 7 days after each vaccination and, additionally, unsolicited AEs, including SAEs, were recorded throughout the duration of the study.

The protocol and study documents were approved by the Institutional Review Boards of the respective study centres - National Hospital Organization Tokyo National Hospital and Haradoi Hospital. The study was conducted in accordance with Good Clinical Practice (GCP) and the Declaration of Helsinki. GSK Biologicals (Wavre, Belgium) sponsored the study and was involved in all stages of the study conduct, including analysis of data. All authors had full access to the data and were involved in the analysis of data and preparation of the manuscript.

### Assessment of immunogenicity

The humoral immune response against the vaccine strain (A/Indonesia/5/2005; Clade 2.1), as well as against the heterologous strains (A/turkey/Turkey/1/2005; Clade 2.2 and A/Vietnam/1194/2004; Clade 1) was measured in terms of the standard HI antibody response according to the guidelines of the Committee for Medicinal Products for Human Use (CHMP) [19]. In addition, neutralising antibodies against the vaccine (A/Indonesia/5/2005) and one heterologous strain (A/Vietnam/1194/2004) were measured by the microneutralisation (MN) assay and are further referred to as H5N1 neutralising antibodies. Studies have shown that neutralisation assays may be more sensitive than the HI test in detecting both greater increases in antibody levels and in detecting infected individuals who are seronegative according to the HI assay [20].

Specific HI antibody titres were determined at GSK Biologicals' laboratories using methods described elsewhere [21]. Antibody titre measurements were conducted on thawed frozen serum samples with a standardised and validated micromethod using four haemagglutination-inhibiting units (HIU) of the appropriate antigens and a 0.5% horse erythrocyte suspension. Non-specific serum inhibitors were removed by subjecting the sera to heat treatment (56°C) and receptor-destroying enzyme. The sera obtained were evaluated for HI antibody levels. Starting with an initial dilution of 1:10, a dilution series (by a factor of 2) was prepared up to an end dilution of 1:20,480. The titration endpoint was taken as the highest dilution step that showed complete inhibition (100%) of haemagglutination. All assays were performed in duplicate.

The titre of H5N1 virus neutralising antibody contained in the serum was determined by an MN assay on thawed frozen serum samples. Each sample was tested in triplicate. Non-specific serum inhibitors were removed by subjecting the sera to heat treatment (56°C). A standardised amount of virus (100 infectious Unit [TCID50] in 0.05 mL) was mixed with serial dilutions of sera and incubated to allow binding of the antibodies to the virus. A cell suspension, containing a defined number of Madin-Darby canine kidney (MDCK) cells was then added to the mixture of virus and antiserum and incubated at 33°C for 7 days. After the incubation period, virus replication was visualised by haemagglutination of chicken red blood cells. The 50% neutralisation titre of a serum was calculated by the method of Reed and Muench [22].

### Assessment of safety

Adverse events were classified according to the Medical Dictionary for Regulatory Activities (MedDRA). The occurrence of AEs was recorded by the subjects themselves using diary cards. In addition, investigators solicited information on specific local AEs (swelling/induration, redness and pain at injection site) and general AEs (fever, headache, fatigue, muscle aches, sweating, joint pain and shivering) occurring within 7 days of each vaccination. Symptom intensity was assessed on a 3-point scale where grade 3 represents the most intense. For both unsolicited AEs and solicited general AEs, the investigators determined the likely relationship of vaccination to symptoms. Intensity and relationship to vaccination of unsolicited local and general AEs were recorded during a 21-day follow-up period from each vaccine administration, as well as overall (Day 0 through to Day 84). All solicited local (injection site) reactions were considered causally related to vaccination. The occurrence of SAEs was recorded during the entire study (up to Day 182).

Haematological and biochemical parameter testing was performed by SRL Medisearch Inc, Japan. The number and percentage of subjects with normal or abnormal haematological and biochemical values, and with normal or abnormal urine values at Day 0, 7 and 42, were calculated. An assessment of these haematological, biochemical and urine parameters was performed at Day 7 and 21 - all parameters were reviewed at Day 7 by the investigators before administering the second vaccine dose at Day 21. Blood parameters assessed were complete blood count, blood urea nitrogen (BUN), creatinine, alanine amino transferase (ALT) and aspartate aminotransferase (AST). Urine parameters measured were protein, glucose, blood and urobilinogen.

### Statistical analysis

The statistical methods for all immunogenicity analyses were performed using the per protocol group. The primary objective of this study was to evaluate the humoral immune response induced by two doses of the H5N1 influenza candidate vaccine in terms of H5N1 HI antibody titres against the vaccine strain. The immunogenicity assessments were based on the surrogate HI endpoints as required by regulatory authorities (CBER and CHMP). In order to meet or exceed these immunogenicity guidance criteria, a target sample size of 100 subjects was required in order to ensure 90 evaluable subjects. Taking into account a 10% drop-out rate and considering a true seroconversion rate (SCR for HI antibodies was defined as the percentage of subjects with either a pre-vaccination titre <1:10 and a post-vaccination titre ≥1:40 or a pre-vaccination titre ≥1:10 and at least a 4-fold increase in post-vaccination titre) of 83.7% and a true seroprotection rate (SPR; defined as the percentage of subjects with a serum H5N1 HI antibody titre ≥1:40) of 84.3%, the proposed sample size allowed for an overall probability of above 85% of meeting the lower limits of 95% confidence intervals (CIs) for SCRs and SPRs of 40% and 70%, respectively. Ninety five percent CIs were calculated for geometric mean titres (GMTs) by exponential transformation of the 95% CI for the mean of log-transformed titres, assuming normal distribution of log-transformed titres.

The immunogenicity analysis was performed for each age stratum and overall. The humoral immune response endpoints in terms of H5N1 HI antibodies were measured using the GMTs of H5N1 HI antibody titres at Day 0, 21, 42 and 182, SCR at Day 21, 42 and Day 182 and seroconversion factors (SCF; defined as the fold increase in serum H5N1 HI antibody GMTs post-vaccination compared with Day 0) at Day 21, 42 and Day 182. The CHMP cut off for SCF is defined as a ratio of >2.5. SPRs were also calculated at Day 0, 21, 42 and 182. Seropositivity was defined as an HI titre >1:10.

For neutralizing antibodies, the endpoints (with 95% CIs) were seropositivity, GMTs and SCRs (SCR for MN antibodies was defined as the percentage of subjects with at least four-fold increase in post-vaccination neutralising antibody titres). The GMTs of neutralising antibody titres were calculated at Day 0, 42 and 182, and the SCRs in terms of neutralising antibody titres were calculated at Day 42 and 182.

The safety analysis was performed on all subjects receiving at least one vaccination (the total vaccinated cohort [TVC]). The percentage of subjects with at least one local AE (solicited and unsolicited), at least one general AE (solicited and unsolicited) and any AE during the solicited follow-up period was tabulated, with exact 95% CI after each vaccination and overall per subject considering both post-immunisation periods.

Solicited symptoms and any pain relief and/or antipyretics taken by the subject to correct the symptoms of local and/or general solicited symptoms were recorded during the 7-day follow-up period after each H5N1 vaccination.

## Results

A total of 100 subjects (n = 50 for 20-40 years of age; n = 50 for 41-64 years of age) were enrolled in September 2008, all of whom received two doses of study vaccine by October 2008. The per protocol cohort was, therefore, identical to the TVC. The mean age of the vaccinated subjects (total cohort) was 40.3 years, 31.1 years for the 20-40 years' stratum and 49.6 years for the 41-64 years' stratum. The overall male-female distribution was 43% versus 57%. The demographic characteristics of the subjects involved are shown in Table 1.

**Table 1:** Demographic data of trial subjects.

		20-40 Y		41-64 Y		Total	
		N = 50		N = 50		N = 100	
**Characteristics**	**Parameters or** **Categories**	**Value** **or n**	**%**	**Value** **or n**	**%**	**Value** **or n**	**%**

Age (years)	Mean	31.1	-	49.6	-	40.3	-
	SD	5.69	-	6.04	-	10.89	-
	Median	31	-	49	-	40.5	-
	Minimum	20	-	41	-	20	-
	Maximum	40	-	63	-	63	-

Gender	Female	25	50	32	64	57	57
	Male	25	50	18	36	43	43

Race	Asian-Japanese heritage	50	100	50	100	100	100

Height (cm)	Mean	165.9	-	162.5	-	164.2	-
	SD	8.41	-	7.46	-	8.1	-
	Median	164	-	161.5	-	163	-

Weight (kg)	Mean	60.7	-	60.6	-	60.6	-
	SD	12.13	-	11.28	-	11.65	-
	Median	61	-	57.6	-	59	-

20-40 Y = subjects aged 20-40 years; 41-64 Y = subjects aged 41-64 years; N = total number of subjects;

n/% = number/percentage of subjects in a given category; value = value of the considered parameter;

SD = standard deviation

### Immunogenicity and cross-clade antibody titres

Only five out of 100 subjects were seropositive against the vaccine-homologous strain (A/Indonesia/5/2005; Clade 2.1) at Day 0 (before vaccination), of whom none had a seroprotective HI titre of 1:40 or more. Pre-vaccination HI GMTs against the other two vaccine-heterologous strains (A/Vietnam/1194/2004; Clade 1 and A/turkey/Turkey/1/2005; Clade 2.2) were also very low and were almost the same as for the vaccine-homologous strain.

Immune responses against the vaccine-homologous strain (A/Indonesia/5/2005; Clade 2.1) at Day 42 fulfilled and exceeded all CHMP and CBER criteria for HI antibody response (Table 2). Overall, the homologous HI SCR and SPR were found to be 91% and were comparable for the predefined age strata (90% for 20-40 years, 92% for 41-64 years). A high cross-reactive (heterologous HI) humoral immune response was observed against the A/turkey/Turkey/1/2005 strain (H5N1 Clade 2.2) and to a lower extent against the A/Vietnam/1194/2004 strain (H5N1 Clade 1) after the second vaccine administration. The HI antibody response against each of the A/turkey/Turkey/1/2005 and A/Vietnam/1194/2004 strains after two doses was similar across the predefined age strata.

**Table 2:** Haemagglutination inhibition (HI) antibody immune responses to homologous and heterologous H5N1 influenza strains following one or two doses of the AS03A-adjuvanted A/Indonesia/5/2005 (H5N1) influenza vaccine were assessed in terms of seropositivity rates, GMTs, SCRs, SCFs and SPRs.

			Seropositivity rates			GMT	SCR *(Negative pre-vaccination HI titre and P/V HI titre ≥1:40, or proportion with ≥4-fold increase from pre- to post vaccination)*			SCF *(Mean GMT increase in titre >2.5 [adults]; >2.0 [aged over 60 y])*		SPR *(% subjects with P/V HI titre ≥1:40)*		
**Antibody**	**Group**	**D**	**N**	**n**	**% (95% CI)**	**Value (95% CI)**	**N**	**n**	**% (95% CI)**	**N**	**Value (95% CI)**	**N**	**n**	**% (95% CI)**

A/Indonesia	20-40 Y	0	50	0	0.0 (0.0-7.1)	5.0 (5.0-5.0)	-	-	-	-	-	50	0	0.0 (0.0-7.1)
		21	50	24	48.0 (33.7-62.6)	15.8 (11.0-22.8)	50	19	38.0 (24.7-52.8)	50	3.2 (2.2-4.6)	50	19	38.0 (24.7-52.8)
		42	50	45	90.0 (78.2-96.7)	156.8 (105.8-232.3)	50	45	90.0 (78.2-96.7)	50	31.4 (21.2-46.5)	50	45	90.0 (78.2-96.7)
		182	49	30	61.2 (46.2-74.8)	25.6 (17.3-38.1)	49	29	59.2 (44.2-73.0)	49	5.1 (3.5-7.6)	49	29	59.2 (44.2-73.0)
														
	41-64 Y	0	50	5	10.0 (3.3-21.8)	5.4 (5.0-5.8)	-	-	-	-	-	50	0	0.0 (0.0-7.1)
		21	50	27	54.0 (39.3-68.2)	15.4 (10.7-22.0)	50	16	32.0 (19.5-46.7)	50	2.8 (2.0-4.0)	50	16	32.0 (19.5-46.7)
		42	50	48	96.0 (86.3-99.5)	142.1 (104.0-194.3)	50	46	92.0 (80.8-97.8)	50	26.2 (19.2-35.8)	50	46	92.0 (80.8-97.8)
		182	50	40	80.0 (66.3-90.0)	37.4 (27.5-50.8)	50	38	76.0 (61.8-86.9)	50	6.9 (5.1-9.2)	50	38	76.0 (61.8-86.9)
														
A/turkey/Turkey	20-40 Y	0	50	2	4.0 (0.5-13.7)	5.7 (4.8-6.8)	-	-	-	-	-	50	2	4.0 (0.5-13.7)
		21	50	13	26.0 (14.6-40.3)	8.0 (6.1-10.4)	50	3	6.0 (1.3-16.5)	50	1.4 (1.1-1.8)	50	5	10.0 (3.3-21.8)
		42	50	30	60.0 (45.2-73.6)	24.8 (16.6-37.1)	50	27	54.0 (39.3-68.2)	50	4.4 (3.0-6.5)	50	29	58.0 (43.2-71.8)
		182	49	30	61.2 (46.2-74.8)	19.2 (13.4-27.3)	49	18	36.7 (23.4-51.7)	49	3.4 (2.4-4.7)	49	20	40.8 (27.0-55.8)
														
	41-64 Y	0	50	2	4.0 (0.5-13.7)	5.2 (4.9-5.5)	-	-	-	-	-	50	0	0.0 (0.0-7.1)
		21	50	18	36.0 (22.9-50.8)	9.6 (7.3-12.5)	50	7	14.0 (5.8-26.7)	50	1.8 (1.4-2.4)	50	8	16.0 (7.2-29.1)
		42	50	31	62.0 (47.2-75.3)	24.0 (16.1-35.6)	50	27	54.0 (39.3-68.2)	50	4.6 (3.1-6.8)	50	27	54.0 (39.3-68.2)
		182	50	38	76.0 (61.8-86.9)	30.7 (22.2-42.5)	50	30	60.0 (45.2-73.6)	50	5.9 (4.2-8.2)	50	30	60.0 (45.2-73.6)
														
A/Vietnam	20-40 Y	0	50	1	2.0 (0.1-10.6)	5.2 (4.8-5.7)	-	-	-	-	-	50	1	2.0 (0.1-10.6)
		21	50	5	10.0 (3.3-21.8)	5.7 (5.0-6.5)	50	0	0 (0-7.1)	50	1.1 (1.0-1.2)	50	1	2.0 (0.1-10.6)
		42	50	26	52.0 (37.4-66.3)	12.7 (9.5-17.1)	50	14	28.0 (16.2-42.5)	50	2.4 (1.8-3.2)	50	15	30.0 (17.9-44.6)
		182	49	17	34.7 (21.7-49.6)	8.6 (6.8-10.8)	49	2	4.1 (0.5-14.0)	49	1.6 (1.3-2.1)	49	3	6.1 (1.3-16.9)
														
	41-64 Y	0	50	5	10.0 (3.3-21.8)	5.9 (5.0-6.8)	-	-	-	-	-	50	2	4.0 (0.5-13.7)
		21	50	13	26.0 (14.6-40.3)	7.0 (5.8-8.5)	50	1	2.0 (0.1-10.6)	50	1.2 (1.1-1.4)	50	4	8.0 (2.2-19.2)
		42	50	24	48.0 (33.7-62.6)	13.3 (9.7-18.2)	50	12	24.0 (13.1-38.2)	50	2.3 (1.7-3.0)	50	15	30.0 (17.9-44.6)
		182	50	23	46.0 (31.8-60.7)	10.7 (8.2-13.9)	50	10	20.0 (10.0-33.7)	50	1.8 (1.4-2.4)	50	12	24.0 (13.1-38.2)

Subjects received one dose of vaccine on Day 0 and one dose on Day 21 (ATP cohort for immunogenicity and persistence).

20-40 Y = subjects aged 20-40 years; 41-64 Y = subjects aged 41-64 years; GMT = geometric mean antibody titre; N = number of subjects with available results; n/% = number/percentage of subjects; P/V = post-vaccination; 95% CI = 95% confidence interval; D = Day, 0 = pre-vaccination, 21 = 21 days post vaccination one, 42 = 42 days post vaccination one (i.e. 21 days post vaccination two), 182 = 182 post vaccination one.

Committee for Medicinal Products for Human Use (CHMP) criteria for adults 18-60 years: SCR >40%, SPR >70% and a SCF of >2.5

US Food and Drug Administration (FDA) Center for Biologics Evaluation and Research (CBER) criteria for adults <65 years of age: Lower bound of the two-sided 95% CI for the percentage of subjects achieving seroconversion for HI antibody should meet or exceed 40% and the lower bound of the two-sided 95% CI for the percentage of subjects achieving a seroprotective level of HI antibody titre ≥1:40 should meet or exceed 70%.

Persistence of the immune response was observed on Day 182 (i.e., 6 months after the first dose); a substantial proportion of subjects (in both age strata) were still seropositive for H5N1 HI antibodies against the A/Indonesia/5/2005 strain (Clade 2.1) and the A/turkey/Turkey/1/2005 strain (Clade 2.2) and, to a lower extent, against the A/Vietnam/1194/2004 strain (Clade 1). The GMTs for H5N1 HI antibodies decreased against all three H5N1 strains compared with Day 42. On Day 182, all three CHMP criteria were still met for H5N1 HI antibody responses against the A/Indonesia/5/2005 strain (except the SPR threshold for subjects aged 20-40 years). Two of the three CHMP criteria (SCR and SCF) for H5N1 antibody response against the A/turkey/Turkey/1/2005 strain were still met in the 41-64 years age stratum.

Trends for higher HI antibody responses were observed against both vaccine homologous and heterologous strains 21 days after the second vaccine dose, in subjects who were seropositive before vaccination. This must be interpreted with caution given the low number of seropositive subjects before vaccination (N = 4 to 6).

### Neutralising antibody response

Neutralising antibody response to the vaccine homologous and heterologous strains at different time points have been presented in Table 3. A marked post-vaccination response in terms of neutralising antibody titres (GMTs) was observed against both Clade 1 and Clade 2 viruses. The observed data suggested that pre-vaccination seropositivity rates against the A/Indonesia/5/2005 strain were low (11.1%) and were higher against the vaccine heterologous strain A/Vietnam/1194/2004 (50%). Following vaccination, the seropositivity rates for neutralising antibody titres against the A/Indonesia/5/2005 strain and the A/Vietnam/1194/2004 strain were increased at Day 42, reaching 100% and 95%, respectively.

**Table 3:** Neutralising antibody immune responses to homologous and heterologous H5N1 influenza strains following one or two doses of the AS03A-adjuvanted A/Indonesia/5/2005 (H5N1) influenza vaccine in terms of seropositivity rates and seroconversion rates.

Antibody	Time point	N	Seropositivity % (95% CI)	N	Seroconversion % (95% CI)
A/Indonesia	PRE	99	11.1 (5.7-19.0)	--	--
	Day 42	100	100 (96.4-100)	99	97.0 (91.4-99.4)
	Day 182	99	100 (96.3-100)	98	93.9 (87.1-97.7)

A/Vietnam	PRE	100	50.0 (39.8-60.2)	--	--
	Day 42	100	95.0 (88.7-98.4)	100	47.0 (36.9-57.2)
	Day 182	99	92.9 (86.0-97.1)	99	58.6 (48.2-68.4)

Subjects received one dose of vaccine on Day 0 and one dose on Day 21 (ATP cohort for immunogenicity and persistence).

Seroconversion rate for MN antibodies: Percentage of subjects with at least four-fold increase post-vaccination neutralising antibody titres.

The SCR for neutralising antibodies increased after the second vaccination and reached 97% and 47% at Day 42 against the A/Indonesia/5/2005 strain and the A/Vietnam/1194/2004 strain, respectively. Six months after the first vaccination, high seropositivity rates were observed in terms of H5N1 neutralising antibodies against both A/Indonesia/5/2005 (100%) and A/Vietnam/1194/2004 (92.9%). The SCR against the A/Indonesia/5/2005 and the A/Vietnam/1194/2004 strains at 6 months were 93.9% and 58.6%, respectively.

### Safety

Compliance in returning safety diary cards was excellent (100%) for both general and local symptoms. There were no SAEs and no withdrawals due to AEs.

**Figure 2:**
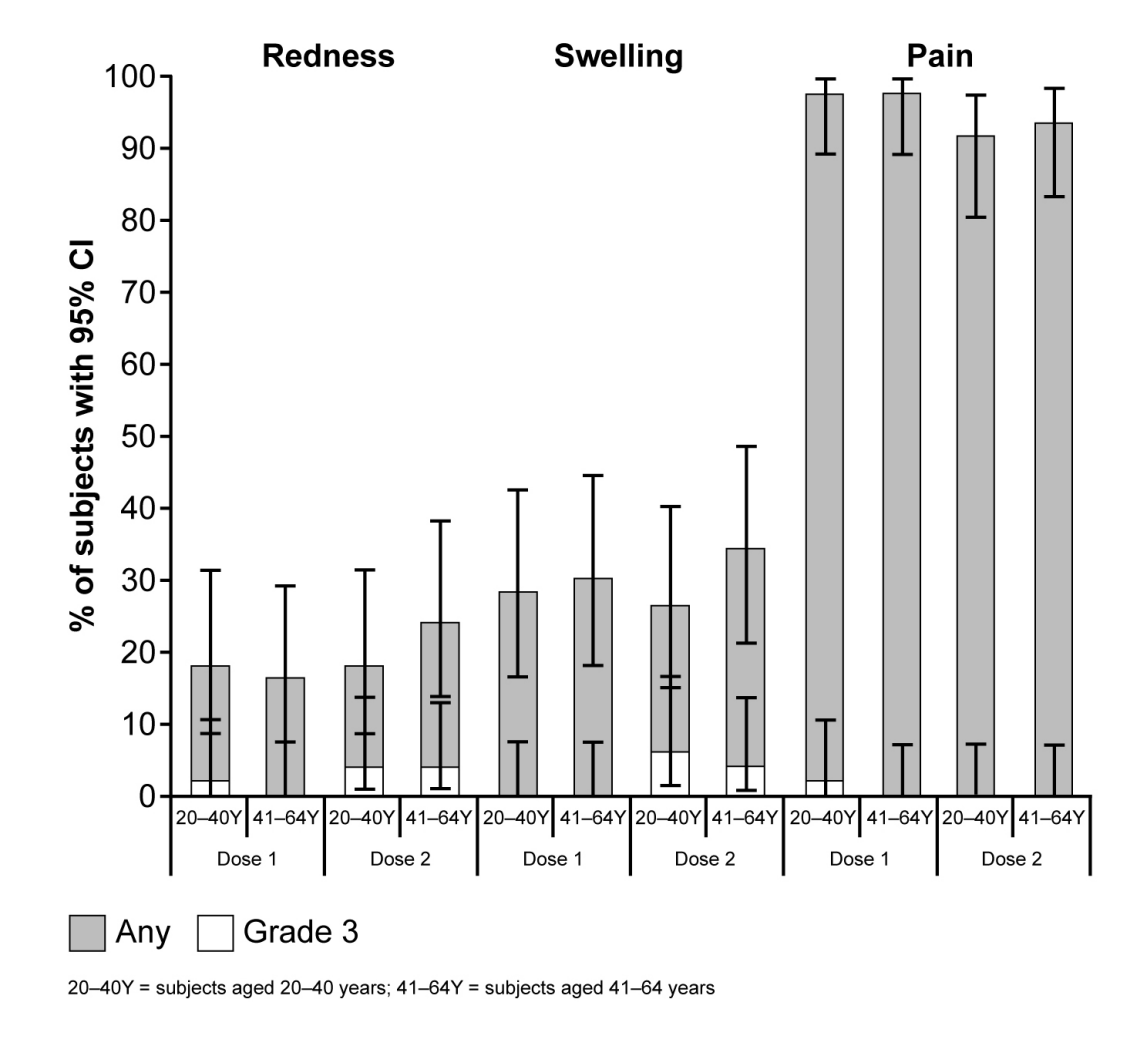
**Solicited local symptoms of any grade and grade 3 severities, following two doses of the AS03_A_-adjuvanted A/Indonesia/5/2005 (H5N1) vaccine, given 21 days apart**.

**Figure 3:**
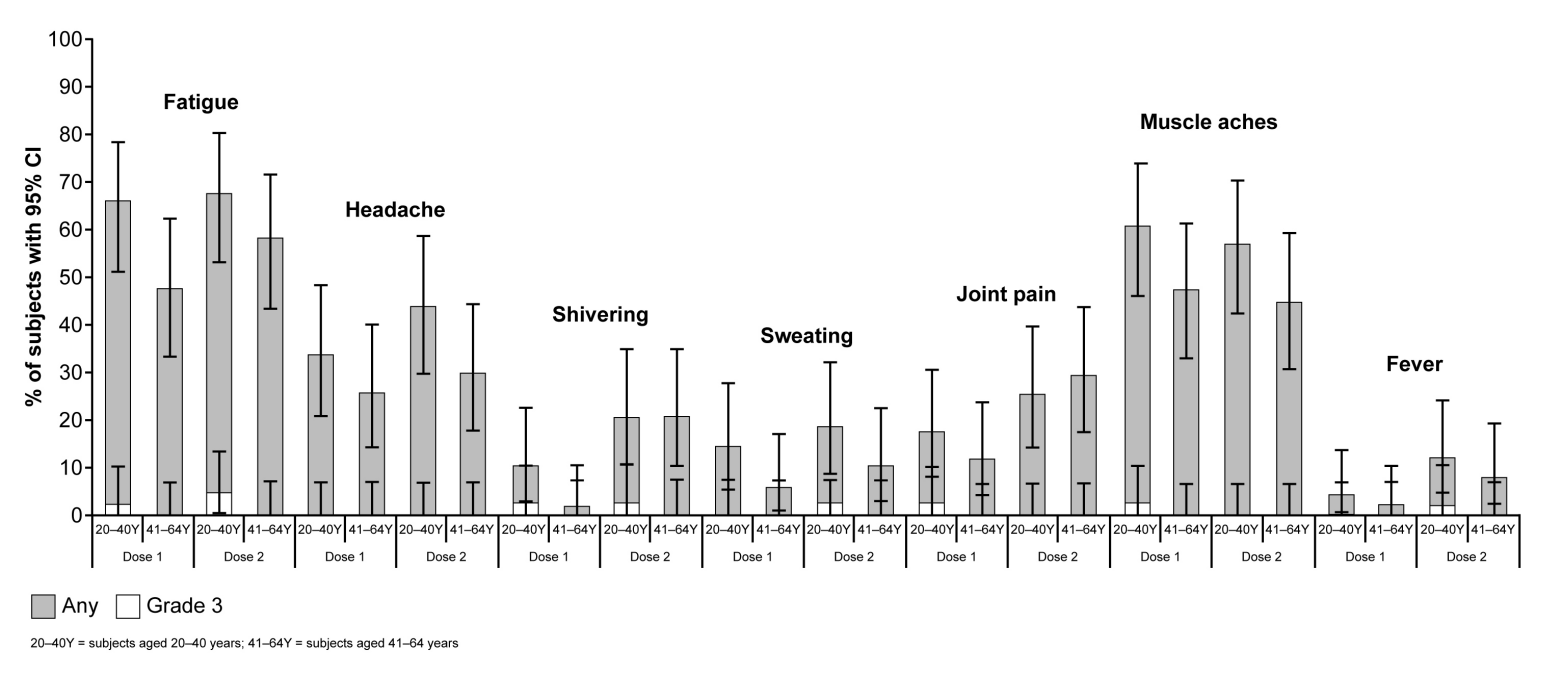
**Solicited general symptoms of any grade and grade 3 severities, following two doses of the AS03_A_-adjuvanted A/Indonesia/5/2005 (H5N1) vaccine, given 21 days apart**.

Solicited local and general AEs are shown in Figures 2 and 3, respectively, and the proportion of subjects reporting fever is shown in Figure 3. Pain at the injection site (of any grade) was the most frequently reported local symptom in the overall cohort after dose 1 (98%) and dose 2 (93%), and there was no observable difference between age strata. The next most frequently observed local symptom was swelling/induration, followed by redness. Grade 3 pain was reported in one subject in the 20-40 years' stratum. General solicited symptoms included fatigue (most common, reported by 71% of subjects overall) followed by muscle aches (70% overall) and headache (51% overall).

Overall, 69% of the subjects reported at least one unsolicited AE, with nasopharyngitis (18%) and injection site pruritus (18%) as the most frequently reported unsolicited AEs in the 20-40 year and 41-64 year cohorts, respectively. There were no major or clinically relevant differences between age strata for any AE and no specific clinical pattern of unsolicited AEs could be identified in either group. Grade 3 unsolicited AEs and grade 3 unsolicited AEs considered related to any vaccination were infrequent and similar in both age strata (20-40 years, three subjects with at least one symptom and one of these symptoms considered by an investigator to be related to vaccination; 41-64 years, three subjects reporting at least one symptom, no related symptoms). No pattern was observed regarding biochemical abnormalities.

## Discussion

The results of this study demonstrate that the AS03_A_-adjuvanted A/Indonesia/5/2005 (H5N1) vaccine was well tolerated and induced strong humoral immune responses in healthy Japanese adults.

The primary endpoints for this study were reached at 21 days after the second vaccination, with immune responses fulfilling all CHMP and CBER criteria for the vaccine-homologous HI antibody response (A/Indonesia/5/2005, Clade 2.1). Overall, homologous HI SCR and SPR were 91% and no differences between the predefined age strata were observed (90% for 20-40 years, 92% for 41-64 years).

These responses were achieved with low doses of antigen (3.75 μg HA). The 'antigen-sparing' effect of the adjuvant AS03 was not demonstrated in this study, as no comparison was made with an unadjuvanted formulation containing 3.75 μg HA; however, the current study is in line with previous influenza vaccine studies with this adjuvant formulation which have shown that the AS03-adjuvanted H5N1 vaccines induce a substantially higher immune response than non-adjuvanted formulations [9], with antigen-sparing properties [10-12]. Therefore, the AS03 adjuvant plays a key role in providing high levels of immunity at relatively low antigen doses, which is one of the requirements for a viable pandemic vaccine in the context of mass distribution.

The H5N1 A/Indonesia/5/2005 vaccine described here was tested in a two-dose regimen with responses to the vaccine strain that met CHMP and CBER criteria only at 21 days after the second injection. This is in contrast to the GSK Biologicals' A/California/7/2009 H1N1 pandemic vaccine adjuvanted with AS03_A _(*Pandemrix*™) which was licensed for use in the 2009/2010 H1N1 pandemic [23]. While posology guidelines published by the European Medicines Agency indicate that a second dose of the vaccine may be desirable to achieve maximum seroprotection, data suggest that a single dose of the H1N1 vaccine may be sufficient to achieve this in healthy adults aged 18-60 years [23]. The reason for this difference may be that naturally acquired partial immunity against A/California/7/2009 (H1N1) is more common, due to prior exposure to circulating H1N1 strains with epitopes similar to those found in A/California/7/2009 (H1N1).

In the current study, SCR and SPR of more than 54% for H5N1 HI antibodies against the Clade 2.2 A/turkey/Turkey/1/2005 were observed after two doses in each age stratum, indicating that the vaccine was markedly cross-immunogenic. This was also observed, albeit to a lesser extent, against the A/Vietnam/1194/2004 (Clade 1) strain, where SCR and SPR of more than 24% were observed. This finding is similar to those of previous studies with the AS03-adjuvanted H5N1 vaccine formulated with the A/Vietnam/1194/2004 (Clade 1) strain where significant levels of cross-clade immunogenicity were observed against antigenically distinct strains of H5N1, including the strains from the other Clades [9,10,12,13]. In a study in Asian adults, following two doses of the AS03-adjuvanted H5N1 vaccine, seroprotection rates in terms of HI antibodies against the vaccine homologous (A/Vietnam/1194/2004) and heterologous (A/Indonesia/5/2005) strains were 94.3% and 50.2%, respectively. For neutralising antibodies against the A/Vietnam/1194/2004 and A/Indonesia/5/2005 (Clade 2.1) strains, seroconversion rates were 96% and 91.4%, respectively [9]. In another study in Europe, two doses of the H5N1 vaccine elicited strong immune responses against vaccine heterologous A/turkey/Turkey/1/2005 (Clade 2.2) and A/Anhui/1/2005 (Clade 2.3) strains (neutralising seroconversion rates: 75- 85%). The study also reported persistence of neutralising seroconversion rates in 60-70% of subjects, up to six months after vaccination [12]. The trend for higher immune responses observed against A/turkey/Turkey/1/2005 may be explained by its lower antigenic distance with the vaccine strain (both are Clade 2 strains) a trend also observed previously [12], in comparison with the A/Vietnam/1194/2004 strain which belongs to Clade 1. H5N1 influenza vaccine efficacy cannot be evaluated in humans for obvious reasons; however, recent studies in a robust ferret model have shown high levels of protection against heterologous challenge following vaccination with a split-virion A/H5N1/Vietnam/1194/04 vaccine formulated with AS03 [15].

Although a substantial proportion of subjects were still seropositive 6 months after vaccination, a reduction of the vaccine response was observed in both age strata for all three strains. However, the data of Schwarz et al. [13] showed effective boosting of immune responses 6 months after primary vaccination. This indicates that the waning response observed at 6 months in this study could be restored by a single booster vaccination with a homologous or heterologous strain.

Moderate levels of baseline seropositivity have already been observed in other populations, particularly for the A/Vietnam strain. In a previous study in a large Asian population, ≤7.2% of subjects was seropositive for the A/Vietnam strain prior to vaccination [9]. The fact that the seropositivity was observed in individuals aged >55 years may concur with repeated exposure to conserved epitopes of seasonal influenza antigens (either through vaccination or natural infection), possibly at the origin of a moderate cross-reactogenic response; this is supported by several reports that showed cross-reactivity between human and avian strains [24-27].

The reactogenicity and safety profiles reported in healthy Japanese adults were similar to those seen in other studies conducted in adults using split-virion, AS03-adjuvanted formulations [9-13], with pain (of any grade) at the injection site being the most common local solicited AE. These findings are in line with previous studies. The safety profile was otherwise unremarkable, with the most common general solicited symptom being fatigue.

The highly pathogenic avian H5N1 has continued to evolve and diversify over the last decade. The H5 Haemagglutinin (HA) gene, which is the target of choice for the adaptive immune response has been conspicuous in its presence in all isolates since 1996. Data available with the WHO indicate that as of March 2009, at least 10 distinct clades have arisen due to genetic re-assortment [28]. A recent modeling study has reported that point mutations in the H5 gene may have led to the evolution of 20 genetically and potentially antigenically distinct strains [29]. The WHO recommends that individual national authorities be consulted and epidemiological and geographical distribution of circulating H5N1 strains be evaluated to decide on the specific H5N1 viruses to be used in H5N1 prepandemic vaccines for respective countries [30]. The vaccine strain (A/Indonesia/5/2005) in this study though first isolated in 2005 was a dominant strain at the time of conduct of this study and was recommended by the WHO for that year; as of February 2010, the WHO has not proposed any new H5N1 strain for vaccine development purposes [30].

## Conclusions

The H5N1 candidate vaccine adjuvanted with AS03_A _elicited a strong and persistent immune response both against the vaccine strain and two strains heterologous to the vaccine in Japanese adults, together with clinically acceptable reactogenicity and safety profiles.

## Abbreviations

AE: Adverse event; AST: Aspartate aminotransferase; ALT: Alanine aminotransferase; ATP: According to protocol; BUN: Blood urea nitrogen; CBER: Center for Biologics Evaluation and Research; CDC: Centers for Disease Control and Prevention; CHMP: Committee for Medicinal Products for Human Use; CI: Confidence interval; FDA: US Food and Drug Administration; GCP: Good Clinical Practice; GMT: Geometric mean titre; HA: Haemagglutinin; HI: Haemagglutination inhibition; HIV: Human immunodeficiency virus; HPV: Human papillomavirus; MDCK: Madin-Darby canine kidney cells; MEDDRA: Medical Dictionary for Regulatory Activities; MN: Microneutralisation; O/W: Oil-in-water; SAE: Serious adverse event; SCF: Seroconversion factor; SCR: Seroconversion rate; SPR: Seroprotection rate; TVC: Total vaccinated cohort; WHO: World Health Organization.

## Competing interests

GSK Biologicals was, in part, the funding source and was involved in all stages of the study conduct and analysis. GSK Biologicals also took charge of all costs associated with the development and publishing of the manuscript. All authors had full access to the data and had the final responsibility to submit the manuscript for publication.

KT, AM, MD and FR are employees of GSK Biologicals. HI has received honoraria/payment for expert testimony and travel grants from the commercial entity which sponsored the study. HN has received honoraria/payment for expert testimony from the commercial entity which sponsored the study.

## Authors' contributions

All authors participated in the design, implementation, analysis and interpretation of the study. All authors read and approved the final manuscript. AM and KT were involved in all phases of the study, and led the clinical team at GSK Japan. HN and HI led the clinical team at their respective centres. MD and FR conducted the data analysis.

## Pre-publication history

The pre-publication history for this paper can be accessed here:

http://www.biomedcentral.com/1471-2334/10/338/prepub
